# Older People’s Use of Digital Technology During the COVID-19 Pandemic

**DOI:** 10.1177/02704676221094731

**Published:** 2022-06

**Authors:** Andrew Sixsmith, Becky R. Horst, Dorina Simeonov, Alex Mihailidis

**Affiliations:** 1 1763 Simon Fraser University, Department of Gerontology; 2 6221 Western University, Department of Neuroscience; 3 488745 AGE-WELL NCE; 4 7938 University of Toronto, AGE-WELL NCE

**Keywords:** information and communication technology, social isolation, digital divide, physical distancing, technology adoption

## Abstract

**Objectives:** The COVID-19 pandemic is having a major impact on the lives of everyone, but in particular on the health and well-being of older people. It has also disrupted the way that individuals access services and interact with one another, and physical distancing and “Stay at Home” orders have seen digital interaction become a necessity. While these restrictions have highlighted the importance of technology in everyday life, little is known about how older adults have responded to this change. **Methods:** Two surveys, one in 2019 and another in 2020 collected data on a combined total of 1923 older adults aged 65 years and older in Canada. These looked at how older adults think about and use technology, with the 2020 survey additionally questioning how COVID-19 has impacted their use and attitudes towards technology. **Results:** While older adults feel more isolated in 2020, many feel positive about the benefits of technology and have increased technology use during the pandemic to support their health, wellness, and communication needs. **Discussion:** The results highlight the potential of technology for supporting older adults in various aspects of healthy aging. While these results point to the opportunities afforded by technology, challenges remain, such as how social and economic factors influence technology uptake.

## Introduction

Globally the COVID-19 pandemic has disrupted the way that individuals access services and interact with one another. Almost all regions have enacted physical distancing or “Stay at Home” orders to restrict in-person interactions to curb the spread of this disease (World Health Organization, 2021). These lockdowns have been a catalyst in which information and communication technologies have become a necessity in order to work, access services, communicate, and participate in leisure activities. Beyond the pandemic, its disruptive nature is likely to have long-lasting implications for how we interact with one another.

Within the first few months of the pandemic, digital technologies saw rapid uptake as digital interaction became a necessity (Kemp, 2020). Internet traffic was 30% higher compared to pre-pandemic levels, use of video conferencing exploded, phone calls per day doubled, and e-commerce was at record highs (Branscombe, 2020). Under stay-at-home restrictions, people's means of social interaction adapted to rely on various technologies. However, these trends may not be equal across different demographic groups, especially older adults who may have additional barriers in using technology (Czaja et al., 2006; Gitlow, 2014).

Prior to the pandemic, previous literature has shown increasing adoption of technology use by older people in recent years (Anderson & Perrin, 2017). More than ever, older adults are using and have access to personal computers, phones, tablets, smart watches, and other devices. In 2017 just over 40% of older adults owned smartphones compared to 18% just three years prior (Anderson & Perrin, 2017). As various technologies become more mainstream, and the internet becomes increasingly engrained within everyday tasks, older adults are likely to also be pushed to more digitally connected lives. Further to this, recent evidence has indicated that the intentional use of various information and communication technologies positively contributes to older adults’ subjective well-being and healthy aging (Nimrod, 2019; Ollevier et al., 2020; Szabo et al., 2019). With the concept of healthy aging including matters such as, staying socially connected, staying physically active, being physically safe, living in place, maintaining independence, and prolonging healthy life.

Yet, historically older adults have been less likely to use technology for everyday services and communication than younger cohorts, due to factors such as digital literacy and access to the internet (Anderson et al., 2021; Hunsaker & Hargittai, 2018). Outside of these factors, personal and environmental context are also important to consider. Older adults are often reported as being resistant to adopting new technologies unless the feelings of usefulness and usability of the technology are greater than feelings of inadequacy (Heinz et al., 2013). In the context of the pandemic, usefulness, and utility of using certain technologies may outweigh the skepticism or hesitancy of use. Additionally, Peek et al. (2016) proposed that psychological and physical contextual factors are paramount to understanding older adult’s perceptions and use of technologies.

Based on previous the results of the literature outlined above, there is strong reason to believe more older adults may be using various information and communication in 2020 compared to the previous year. However, there is limited evidence on how older adults have actually adapted to this greater reliance on technology during the pandemic and whether this context has influenced older adult everyday technology use. In this paper, we examine older people’s use and attitudes towards technology pre-pandemic and three months into the global pandemic and discuss the longer-term implications for health and social policy.

## Research Design

Participant recruitment and data collection were completed by Environics Research (https://environicsanalytics.com/en-ca/about), a North American commercial data analytics and research company. The data collection services provided by Environics was commissioned by AGE-WELL NCE (www.agewell-nce.ca) a federally-funded Networks of Centres of Excellence program in Canada. AGE-WELL NCE focuses on research and innovation to support older adults and caregivers across multiple social and economic challenges. Data collection was outsourced to a larger research body in order to collect a larger and more representative sample than what would be internally feasible. Environics and AGE-WELL are separate research entities and are not related. The collected data was provided to the authors for secondary analysis and approved by the Research Ethics Board at Simon Fraser University REB #30000195.

Two independent cross-sectional surveys were conducted in late June 2019 and late June 2020. Canadian adults over the age of 50, within Environics’ previous participant database and new recruitment reach, were invited to complete the survey and answer questions on their attitudes and behaviors concerning technology. The surveys were offered in both French and English and responses were collected online, or via computer-assisted telephone interview (CATI). Specific quotas were outlined for each survey to ensure, 1) at least 45% of the responses collected were over the age of 65 years old; 2) 51% of the sample was female, and 3) the distribution of geographical responses aligned with proportional representation of the population. For our analysis, only the data of those aged 65 or older was processed.

Survey questions addressed participants’ perceptions towards technology, beliefs that technology can assist with aspects of healthy-aging, device ownership and frequency of use, use of technology for communication, and use of technology for health and wellness. The 2020 version of the survey was identical to the 2019 version but included additional questions to evaluate how COVID-19 had impacted personal technology use and attitudes. These COVID-19 related included questions that explicitly asked if the COVID-19 pandemic has “increased”, “decreased” or had “no change” on their use of the specified technologies and digital services.

Responses in the 2019 survey were compared to the 2020 survey using Chi-squared testing (α = .05). Significant associations were further evaluated using Bonferroni corrected pairwise z-testing, where appropriate, to evaluate where proportions differed.

## Results

The sample included 1923 total responses from Canadians aged over 65, 996 (777 aged 65 to 74 and 219 aged 75 or older) from the 2019 survey and 927 (631 aged 65 to 74 and 296 aged 75 or older) from the 2020 survey. Chi-squared testing to compare demographic characteristics between the two survey years indicated that there were no significant differences in demographic characteristics between the two samples.

### Attitudes Towards Aging and Technology

**Figure 1.**
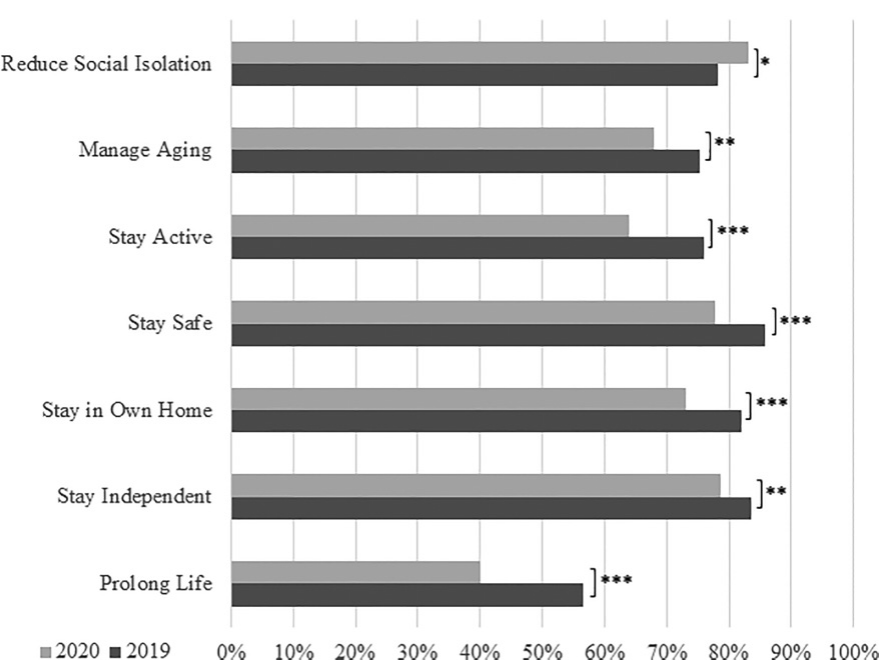
Differences in older adults reported agreement that technological advancements can help with areas of aging, 2019 responses compared to 2020. Differences between groups are tested with Chi-square tests. *Note.* * *p* < .05, ** *p* < .01, *** *p* < .001.

When asked about feelings of isolation, participants in 2020 indicated significantly more feelings of isolation “some of the time” or “often” compared to 2019 (*X*^2^ = 236.05, df = 2, *p* < .001). When questioned about to what extent they agreed or disagreed that technological advancements could help with aspects of healthy aging, mixed results were found (Figure 1). Significantly fewer older adults in 2020 compared to 2019 agreed that advancements in technology can help in all questioned areas, with the exception of reducing social isolation where there was a significantly greater proportion of those who agreed in 2020 (*p* < .05).

### Differences in Technology Use Between 2019 and 2020 Samples

Slightly more older adults in the 2020 sample reported using the internet on a daily basis compared to 2019 (88% in 2020, 86% in 2019), however, this difference was not significant. Only when the group is stratified into younger (65 to 74) and older (75 + ) cohorts did significant differences appear, with significantly more older adults aged 75 and older in 2020 reporting using the internet daily (79.4%) compared to 2019 (71.2%, *X*^2^ = 4.584, df = 1, *p* = .032).

The proportion of older adults reporting ownership of smartphones significantly larger, with 65.3% reporting ownership in 2020, compared to 57.6% in 2019 (*p* < .001). When analyzed by age group, only the difference in the 65 to 75 age group was significant (62.0% in 2019, 72.1% in 2020, *X*^2^ = 15.875, df = 1, *p* < .001). Though the 75 year or older group also demonstrated differences (42.0% up to 50.7% in 2020) it was not significant (*p* = .051). Ownership between the years for other surveyed devices (desktops, laptop, tablet, video game console, wearable device, voice assistant device, or assistive robot) saw more ownership in 2020 but was not significantly different. Reported ownership of certain digital devices was significantly less in 2020 compared to 2019, specifically GPS Devices (46.8% to 36.1%, *p* < .001), non-smart cell phones (23.9% to 17.0%, *p* = .001), and digital cameras (42.5% to 36.1%, *p* = .017).

The proportion of older adults in 2020 who reported using specific devices daily was generally larger in 2020 by one to six percent compared to 2019, however, these differences were not significant. The only significant differences were the use of internet-enabled TVs, where 47.7% of older adults reported using daily compared to 31.3% in 2019 (*X*^2^ = 12.966, df = 1, *p* < .001), and wearable health devices which 80.1% reported using daily in 2020 compared to 69.4% in 2019 (*X*^2^ = 4.489, df = 1, *p* = .034). GPS devices, video game consoles, and digital cameras were the only digital devices for which daily usage compared between the years had decreased (non-significant).

**Figure 2.**
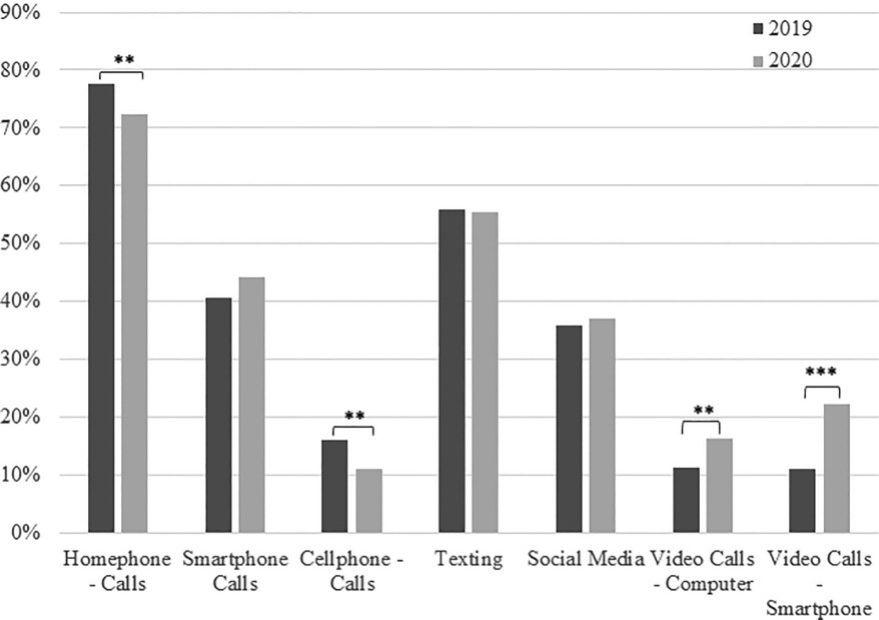
Proportion of participant responses in 2019 and 2020 who use certain digital communication methods to communicate with family and friends. *Note.* * *p* < .05, ** *p* < .01, *** *p* < .001.

Reported modes of communication used to communicate with family and friends in 2020 changed compared to 2019 (Figure 2). The proportion of older adults reporting using a home phone or traditional landline was significantly less in 2020 compared to 2019 (72.3% versus 77.5%, *X*^2^ = 7.012, df = 1, *p* = .008). Use of non-smart cell phones was significantly less (16.0% down to 11.0% in 2020, *X*^2^ = 10.072, df = 1, *p* = .002). The use of phone calls via smartphones was greater, but was not significant. Communication by means of video calls via computer (*X*^2^ = 9.51, df = 1, *p* = .002) or smartphone (*X*^2^ = 43.524, df = 1, *p* < .001) was significantly greater in 2020 (16.2% and 22.3%) compared to 2019 (11.3% and 11.1%).

### Technology Behaviors and Attitudes in Relation to 2020 Survey COVID-19 Questions

When asked about how “Technological advancements could help to lessen the impact of COVID-19 on daily life” 64% of participants in the 2020 survey agreed that technology advancements could help lessen the impact of COVID-19, 9% disagreed, and 27% neither agreed nor disagreed. Older adults in the 65 to 74 years age stratification were more likely to agree than those 75 plus (*X*^2^ = 13.736, df = 2, *p* < .001).

**Figure 3.**
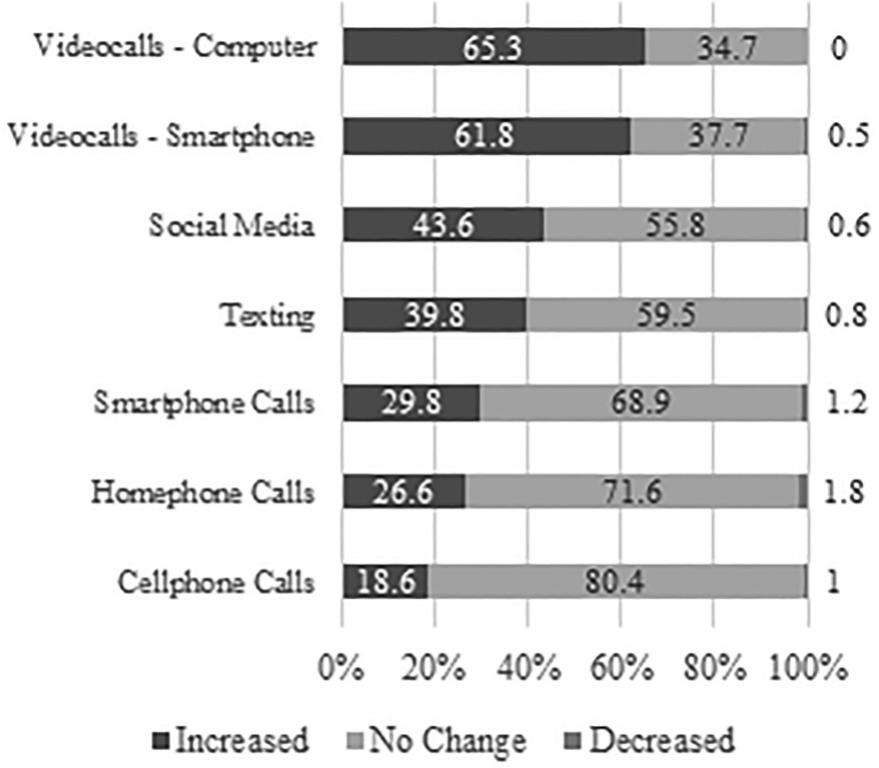
Changes in older adult’s digital communication behaviors to communicate with family and friends due to the COVID-19 pandemic.

When the 2020 sample was asked how COVID-19 had impacted their use of methods to communicate with family and friends, increases were reported (Figure 3). 65.3% and 61.8% of older adults reported using video calls on computers and smartphones more often, followed by increases in social media (43.6%), texting (39.8%), smartphone calls (29.8%), home phone calls (26.6%), and cellphone calls (18.6%). There were no differences between age groups in respect to communication methods, with the exception of computer-video calls, where the 65 to 74-year-olds were more likely to report increases due to COVID-19 (*X*^2^ = 4.594, df = 1, *p* = .032).

The use of specific technology to support health and daily activities had reported changes due to the pandemic. Many respondents reported increases in their digital services use, online shopping: essential items (61%), webinars (51%), online social activities (50%), online shopping: non-essential items (48%), online streaming (46%), food delivery: not groceries (45%), social media (42%), networking apps (42%), physical activity tracker (19%), voice-assisted technology (18%), meditation app (18%), task scheduler (17%), wearable device (17%), online financial tools (16%), and smart home tech (15%). Most older adults (>68%) also reported they will continue to use these technologies post-pandemic. There were no significant relationships between stratified age groups and behavior changes.

## Discussion

Our study showed significant changes in technology-related behaviors and attitudes between 2019 to 2020. The results further support that some of these differences in technology behaviors may be related to the pandemic. Due to restrictions during the pandemic, sweeping transitions of services from in-person to digital media occurred. Our results indicate that older adults in 2020 modified their behaviors accordingly, with higher levels of technology use for everyday activities such as shopping, socializing, and entertainment, compared to 2019. Many older adults reported that they will continue to use these technologies after the pandemic.

Our findings also challenge the stereotype that older adults are unable or unwilling to use digital technologies, with many adopting or increasing technology use for their communication needs in 2020. Our results indicate that even prior to the observed increases in 2020 many older adults in 2019 engaged with and used technology for a variety of everyday purposes. While the numbers of older adults using the internet daily or owning a smartphone device are still lower than younger cohorts (Anderson & Perrin, 2017; Zickuhr & Madden, 2012), our results indicate that a large number of older adults own and use a variety of digital devices on a daily basis.

With notably, a significant increase in usage by the older cohort (75 + ) in certain technology behaviors, particularly daily internet use. This difference may be due to a saturation-like effect of the younger cohort already using internet daily, such that increases due to the pandemic were not significant in the combined and younger age groups.

The longer-term implications of the pandemic are yet to be realized, but in the short term, it appears many older adults have adapted to an increasingly digital world. Our results align with global trends of increased digital communication use during the pandemic, with many older adults adopting and using digital tools to communicate with others. Notably, the older cohort, those aged 75 and older, saw similar or equal increases in technology use as the younger cohort under pandemic restrictions, despite being an age group that typically reports slower uptake and less use of technology in general (Anderson & Perrin, 2017; Davidson & Schimmele, 2019; Hülür & Macdonald, 2020; Mariano et al., 2021; Siren & Grønborg Knudsen, 2017). It should also be noted that the 2020 survey was conducted relatively soon after the global pandemic declaration, at a time when many did not expect restrictions to be prevalent over a year later. This context should be considered as it may have influenced how individuals responded to the rate of uptake or consideration in continuing with certain technologies.

Looking beyond the pandemic, our results indicate that digital technologies could afford opportunities for supporting the well-being of older adults, as many older adults agree that technological advancements can be beneficial for areas of healthy aging, albeit less accepting than the previous year’s cohort. For example, more older adults in 2020 agreed that technology can be used to help combat social isolation, which is known to lead to stress, depression, and poor health outcomes (Coyle & Dugan, 2012). Increases in technology use and reported speculation of continued use should be leveraged to support older adults with components of healthy aging. Refining and using co-design principles on existing technologies that older adults are using during the pandemic could lead to higher levels of engagement and uptake of other older adults to support their wellness and independence (Botero and Hyysalo, 2013; Wherton et al., 2015). An additional follow-up study would be useful in confirming if older adults were indeed honest in their speculation of continued technology use, or if they returned to previous habits once restrictions were removed. Further investigation is also needed to examine why that rate of agreement concerning most examples of technology advancements for healthy aging was lower in 2020 than 2019. Even though the overall majority of older adults had some level of agreement that technology advancements could help with areas of healthy aging, and the majority of older adults felt that technology could help lessen the impact of covid-19, there was a significantly smaller proportion of older adults who agreed with the statements related to healthy aging in 2020 compared to the 2019 sample. We postulate that this decline could be due to increased pessimism related to the pandemic and facing the realities of current technology limitations. However, due to the nature of the survey this cannot be confirmed without additional follow-up.

Critically, our study highlights the differences in older adult’s technology use between two comparable samples, yet definitive evidence that these differences may be due specifically to the COVID-19 pandemic is uncertain. The general trend of technology uptake by older adults has risen over the past years (Anderson et al., 2021), and without longer-term data, we are unable to discern whether 2020 was a significant spike in uptake or in line with previous years. Our data suggests that many older adults increased their technology use because of COVID-19, but directly comparable data would make this conclusion stronger. Regardless, these results are a timely reminder that many older adults are accessing technologies for various reasons and further lends evidence that technology can be used in real-world decisions to support older adults.

Our study is not without limitations. The distribution method of this survey was open to all participants within the Environics membership database, however recruitment may have been biased towards individuals who had strong opinions concerns technology, as this was the recruitment topic for the survey. Even though the 2019 and 2020 samples were comparable populations, the conclusions drawn must consider older adults who may not have been reached through Environic’s survey recruitment.

While our results suggest many positives and opportunities of technology, there are still challenges to consider, such as how social and economic factors create a digital divide that influences technology uptake, and how external factors, such as lack of infrastructure, internet access and low digital literacy, may prevent older adults from accessing technologies (Fang et al., 2019). These factors warrant further and more in-depth investigation outside of what the scope of our survey was able to assess.
